# Characterization of Matricellular Protein Expression Signatures in Mechanistically Diverse Mouse Models of Kidney Injury

**DOI:** 10.1038/s41598-019-52961-5

**Published:** 2019-11-13

**Authors:** Daniel Feng, Cindy Ngov, Nathalie Henley, Nadia Boufaied, Casimiro Gerarduzzi

**Affiliations:** 1 Département de Pharmacologie et Physiologie, Faculté de Médecine, Université de Montréal, Montréal, Québec Canada; 2 Centre de recherche de l’Hôpital Maisonneuve-Rosemont, Faculté de Médecine, Centre affilié à l’Université de Montréal, Montréal, Québec Canada; 3 Department of Microbiology and Immunology, McGill University Health Centre Research Institute, Montréal, Québec Canada; 4 Division of Urology and Cancer Research Program, McGill University Health Centre Research Institute, Montréal, Québec Canada; 5 Département de Médecine, Faculté de Médecine, Université de Montréal, Montréal, Québec Canada

**Keywords:** Data mining, Literature mining, Chronic kidney disease, Renal fibrosis

## Abstract

Fibrosis is the most common pathophysiological manifestation of Chronic Kidney Disease (CKD). It is defined as excessive deposition of extracellular matrix (ECM) proteins. Embedded within the ECM are a family of proteins called Matricellular Proteins (MCPs), which are typically expressed during chronic pathologies for ECM processing. As such, identifying potential MCPs in the pathological secretome of a damaged kidney could serve as diagnostic/therapeutic targets of fibrosis. Using published RNA-Seq data from two kidney injury mouse models of different etiologies, Folic Acid (FA) and Unilateral Ureteral Obstruction (UUO), we compared and contrasted the expression profile of various members from well-known MCP families during the Acute and Fibrotic injury phases. As a result, we identified common and distinct MCP expression signatures between both injury models. Bioinformatic analysis of their differentially expressed MCP genes revealed similar top annotation clusters from Molecular Function and Biological Process networks, which are those commonly involved in fibrosis. Using kidney lysates from FA- and UUO-injured mice, we selected MCP genes from our candidate list to confirm mRNA expression by Western Blot, which correlated with injury progression. Understanding the expressions of MCPs will provide important insight into the processes of kidney repair, and may validate MCPs as biomarkers and/or therapeutic targets of CKD.

## Introduction

Chronic kidney disease (CKD) is a major health concern affecting approximately 10% of the global population^1^. Fibrosis is a maladaptive condition of repair associated with a majority of CKDs. It is characterized by aberrantly excessive accumulation and processing of stiff extracellular matrix (ECM) materials that progressively replaces the flexible parenchymal tissue^2^. In other words, the process of constructive restoration associated with the reparative ECM can shift towards a destructive remodeling (stiffening) of the tissue, leading to permanent scarring, organ malfunction and, ultimately, death^3^. While methods of intervention such as dialysis and transplantation serve as renal replacement therapies, they do not impede the progression of fibrosis. Furthermore, no treatments are currently available to effectively inhibit nor reverse the injury signals associated with maladaptive repair. Indeed, there is an urgent need to study the mechanisms of repair prior to fibrosis onset in order to identify novel biomarkers for disease monitoring as well as therapeutic targets for intervention.

The status of the ECM is a major determinant in the regulation of signaling pathways that drive repair. As such, ECM processing and cell-ECM interactions are important factors to consider in the context of fibrosis. Over the last 20 years, Matricellular Proteins (MCPs) have emerged as essential regulators of these ECM events, and therefore of ECM remodeling and cellular behavior. As secreted proteins, the diverse activities of MCPs are determined by their multiple functional regions that interact with various ECM and cell surface molecules. The dynamic regulatory roles between the cell and its surrounding environment have distinguished MCPs from the “classical” role of ECM proteins as static structural components. In contrast to the continuous presence of ECM proteins in the cellular environment, MCP expression is tightly regulated and transiently expressed to exhibit context-specific effects during normal tissue remodeling processes (i.e. repair)^4,5^. However, MCP expression is sustained during various injuries and chronic diseases, localizing in the extracellular space of diseased tissue and contributing to their pathologies (i.e. fibrosis)^6^. Thus, MCP extracellular location and involvement during disease progression imply their potential to enter into the circulation and serve as non-invasive biomarkers of repair, as well as accessible targets to treat fibrosis with fewer side effects.

The majority of MCPs have been previously characterized in fibrosis; however, only a few have been studied in the context of kidney fibrosis. Despite this progress from numerous experiments focusing on single MCP molecules, global transcriptional profiles of multiple members from major MCP families have not been compiled. Therefore, our goal was to analyze the temporal RNA expression patterns of MCPs during the acute injury phase, and identify those whose expression is sustained during fibrotic pathologies. Specifically, we exploited RNA sequencing (RNA-Seq) data from a toxicant and surgical mouse model of renal injury to screen all known members from the well-characterized MCP families: **S**ecreted **P**rotein **A**cidic and **R**ich in **C**ysteine (**SPARC**), **CCN**, Thrombospondin (**THBS**), **S**mall **I**ntegrin-**B**inding **Li**gand **N**-linked **G**lycoprotein (**SIBLING**) and Tenascin (**TN**). We interrogated the possibility of a conserved MCP transcriptome between these mechanistically different models of injury, and whether a specific type of injury can stimulate its own pattern of MCP expression during the course of repair. In addition, based on MCP genes that are differentially expressed, we predict their respective functions in the two experimental models through bioinformatics analysis. Finally, we confirm the mRNA expression differences of a selection of early and late MCP genes from both mouse models of kidney injury by Western Blot analysis.

Our analysis provides new insight into the plasticity of MCP expression throughout the tissue remodeling process in two mechanistically different injury models, and highlights the potential of these injury-specific proteins as diagnostic/prognostic biomarkers and therapeutic targets of fibrosis, for which there is currently no approved treatment.

## Results

### Defining our MCP candidates and mouse injury models

Over the years, many MCPs have been discovered and placed within distinct families based on shared structure/function^7,8^. The MCP families SPARC, CCN, THBS, SIBLING and TN have been well-studied for their important mechanistic roles in repair. From among these 5 families, a total of 29 members have so far been identified. We believe that the expression profiles of MCP family members with known function will reflect processes occurring at different stages of kidney repair, and/or fibrosis. Moreover, this information can be used to infer the roles of co-members with no known functions in fibrosis. Therefore, we decided to screen all 29 known members belonging to the 5 MCP families. Using PubMed and Kidney and Urinary Pathway Knowledge Base search engines with inclusive terms “Fibrosis” and “Kidney Fibrosis”, we classified each member into the categories of “Known in Kidney Fibrosis”, “Novel to Kidney Fibrosis” or “Novel to Fibrosis” (Table 1).

**Table 1:** Listing of candidate MCP genes based on published fibrotic relevance within *in vitro* and/or *in vivo* models.

	Known in Kidney Fibrosis	Novel to Kidney Fibrosis	Novel to Fibrosis
SPARC family	Sparc	Sparcl1	Spock1
	Smoc2	Smoc1	Spock2
	Fstl1		Spock3
Thrombospondin family	Thbs1	Thbs2	
	Comp	Thbs3	
		Thbs4	
Tenascin family	Tnc		TnxA
			TnxB
			Tnr
			Tnn
CCN family	Cyr61	Wisp2	
	Ctgf		
	Nov		
	Wisp1		
	Wisp3		
Sibling family	Spp1		Bsp
			Dmp1
			Dspp
			Mepe

**Figure 1:**
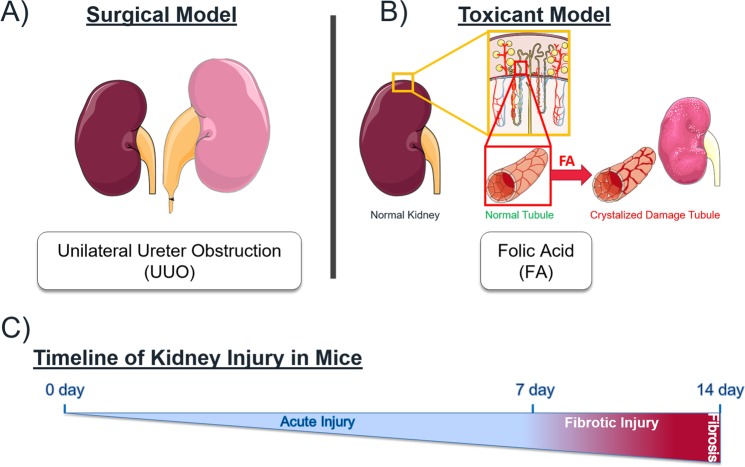
Mechanistically distinct mouse models of kidney injury. **(A)** Unilateral Ureteral Obstruction by surgically tying the urinary tract leading to tubular atrophy, interstitial fibrosis and inflammation. (**B)** Folic Acid administration by intraperitoneal injection results in folic acid crystal formation within the renal tubules with subsequent acute tubular necrosis/apoptosis, inflammatory cell infiltration, tubular cell proliferation, epithelial regeneration and mild fibrosis in the chronic phase. (**C)** Timeline of most persistent injuries which includes an Acute and Fibrotic injury. Figure was produced using Servier Medical Art (http://smart.servier.com/).

We initially exploited two recently published gene expression (RNA-Seq) datasets from injured mouse kidney tissue, each representing a mechanistically different model of injury (Fig. 1), i.e. a Folic Acid (FA) toxicant model, and a Unilateral Ureteral Obstruction (UUO) surgical model. As reliable kidney fibrosis models^9,10^, FA is a regressive injury that permits tissue recovery, while UUO is progressive and irreversible; hence, the former has a more irregular dispersion of fibrotic tissue while the latter exhibits a more robust fibrotic response affecting the whole kidney. For the FA model, the RNA-Seq data which we analyzed was performed on total RNA from triplicate mouse kidney samples harvested at days 0, 1, 2, 3, 7 and 14 post-FA treatment (GEO# GSE65267)^11^. As for the UUO-model, RNA-Seq was performed on total RNA obtained from triplicate kidney samples after 2- and 8-days post-UUO, which was compared to 4 sham operated mice (GEO# GSE79443)^12^. Both injury models typically use 7-days post-injury as a reference point to distinguish between an acute adaptive repair response (<7-days, appearance of provisional and repaired matrix in healing process^13^) and a fibrotic outcome (≥7-days, pathological levels of repair markers^11,14–17^), designated “Acute” and “Fibrotic” phases, respectively. The gene expression levels among our panel of MCPs were analyzed temporally over the course of each injury stimulus, in order to compare similarities and differences between both injuries of distinct etiology.

### Identification of MCP gene expression profiles in folic acid injured kidney

**Figure 2:**
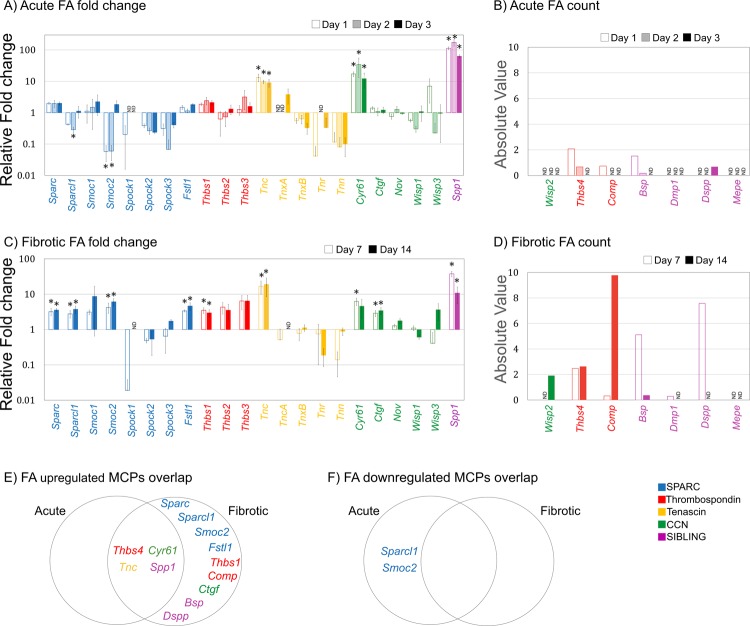
MCP expression in mouse kidney at Acute and Fibrotic time points after FA treatment. RNA-Seq fold change (relative to untreated) and absolute value of MCP mRNA expression from mouse kidneys following a single intraperitoneal injection of 250 mg/kg FA: (**A)** Fold change and (**B)** absolute value of mRNA expression from candidate MCPs at Acute injury time points 1-, 2- and 3-days following injection. (**C)** Fold change and (**D)** absolute value of mRNA expression from candidate MCPs at Fibrotic injury time points 7- and 14-days following injection. *DESeq p ≤ 0.05, n = 3 mice. Venn diagram of MCPs that are differentially (**E)** upregulated and (**F)** downregulated between Acute injury (1-, 2- and 3-days) and Fibrotic injury (7- and 14-days).

FA nephropathy is a simple kidney injury model commonly performed on rodents with injury progression and fibrosis comparable to human kidney diseases^14,18,19^. Intraperitoneal injection (i.p.) of a high dosage of FA is known to accumulate and crystalize within the tubules of the kidney but also to have a direct toxic effect on the epithelial cells. Consequently, this results in tubular lesions that elicit a repair response^20^, accompanied by mild fibrosis at later stages. This development of fibrosis is similar to that found in patients transitioning to CKD, suggesting the fibrotic relevance of the FA-injury model to the human condition^11,21,22^. Consistent with the FA model^11,23,24^, the RNA-Seq dataset exhibited expected changes in gene expressions at the appropriate times for Acute (KIM-1, TGFβ at days 1–3) and Fibrotic (collagen, fibronectin, α-sma at days 7 and 14) injury^11^. Using this time frame, we then evaluated the average fold change (relative to untreated) and absolute expression of our 29 MCP candidate genes to identify those that were up- or down-regulated at Acute and Fibrotic time points of FA-induced injury (Fig. 2A–D). Absolute expression was reserved for genes whose control had no detectable expression (Fig. 2B,D).

We observed distinct dynamic and temporal trends of gene expression between MCP families, but also between MCP family members. Considering the trend of expression during Acute FA-induced injury (days 1–3), SIBLING and THBS genes were mostly upregulated, CCN and TN genes had mixed expression, and SPARC genes were mostly downregulated (Fig. 2A,B). The SIBLING gene *Spp1* was the most upregulated MCP, maintaining high expression over 3-days, and peaking at over 100-fold, while the TN gene *Tnr* was the most downregulated. THBS and SIBLING were the only families with members manifesting expression only after FA injury; hence, their absolute values were used to show that the THBS genes *Thbs4* and *Comp*, and the SIBLING genes *Bsp* (alternatively known as *Ibsp*) and *Dspp*, were upregulated at some point during the 3-days of FA treatment (Fig. 2B). However, the CCN gene *Wisp2*, and SIBLING genes *Dmp1* and *Mepe*, had no detectable expression during the early stages of repair. The gene expression trend for MCPs was further examined during FA-induced Fibrotic injury (days 7 and 14; Fig. 2C,D). SIBLING and THBS family members showed expression levels that were only upregulated, while CCN and SPARC family members were mostly upregulated. The TN genes tended to exhibit downregulated expression except for one member, *Tnc*, which was the second highest expressed MCP next to *Spp1*. The most downregulated MCP gene was from the SPARC family member *Spock1*, which was nearly 90-fold lower. Of the nondetectable MCP genes from normal tissue, *Wisp2* and *Dmp1* presented a negligible increase in expression while *Mepe* still remained undetectable during late injury compared to earlier time points.

Schematically representing the similarities between Acute vs Fibrotic MCP responses, gene expression levels were classified as differentially expressed (Fig. 2E,F) when they were statistically different for at least one time point using DESeq analysis with an adjusted p-value ≤ 0.05 and log2 fold-change > 1.5 (Fig. 2A,C). Included in this analysis were those MCP genes with a detectable expression only after injury with an absolute cut-off FPKM value of >2 (Fig. 2B,D). Of the 29 MCP genes, 13 were differentially upregulated, with none specific to Acute, 9 specific to Fibrotic and 4 in both phases, while 2 genes were differentially downregulated only during the Acute phase. Most of the SPARC family genes were differentially expressed during the Fibrotic MCP response (*Sparcl1, Smoc2, Sparc, Fstl1*). Though upregulation of *Sparcl1* and *Smoc2* genes was classified as a Fibrotic response (Fig. 2E), their expression was initially differentially downregulated during the Acute response (Fig. 2F). *Thbs4, Tnc, Cyr61 and Spp1* were the only genes with at least one time point showing differential expression in both the Acute and Fibrotic phase. The trend of gene expression from an Acute to Fibrotic response was generally increasing for SPARC, THBS and TN families while fairly constant for the CCN family. The SIBLING family had fluctuating expression levels within both time responses, but also the only one with a member, *Mepe*, that did not have any detectable expression at any time point.

### Identification of MCP gene expression profiles in unilateral ureteral obstruction injured kidney

UUO is an established mouse model of interstitial fibrosis that progressively develops from a surgical ligation of the ureter, and consequent interstitial inflammation, tubular dilation, atrophy and apoptosis^16,17^. UUO is not a usual cause of human renal disease but a robust model of hydronephrosis^11^. Furthermore, the UUO model is well-described to recapitulate the fundamental pathological mechanisms that characterizes the various forms of CKD^9^. Gene expression changes of the repair transcriptome had the appearance of Acute injury at day 2 while Fibrosis at day 8^12^. Similar to our FA analysis, we evaluated the average fold change (relative to the control) and absolute expression of each 29 MCP family members within the UUO injury model to characterize them into the Acute and/or Fibrotic phase(s).

**Figure 3:**
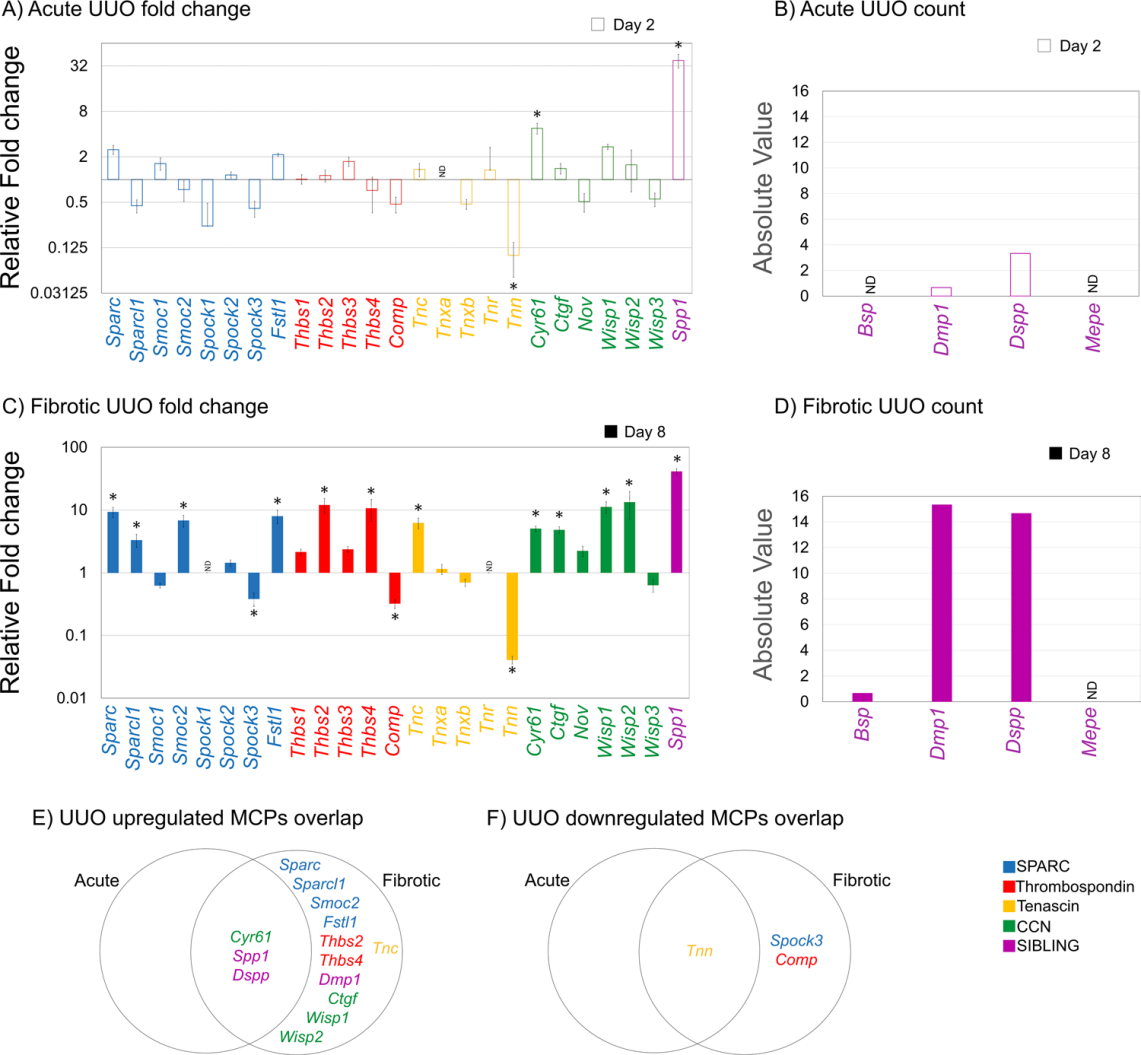
MCP expression in mouse kidney at Acute and Fibrotic time points after UUO surgery. RNA-Seq fold change (relative to the control) and absolute value of MCP mRNA expression from mouse kidneys following UUO surgery: (**A**) Fold change and (**B**) absolute value of mRNA expression from candidate MCPs at Acute injury time points 2-days following surgery. (**C**) Fold change and (**D**) absolute value of mRNA expression from candidate MCPs at Fibrotic injury time points 8-days following surgery. *DESeq p ≤ 0.05, n = 3 mice. Venn diagram of MCPs that are differentially (**E**) upregulated and (**F**) downregulated between Acute injury (2-days) and Fibrotic injury (8-days).

Acute analysis of the MCP gene expression profile from a post-UUO injury (day 2, Fig. 3A,B) indicates variability between families. Members from the SIBLING family had only upregulated gene expressions, including *Spp1*, the highest amongst all MCPs. The only MCPs with undetectable expression were *Tnxa, Bsp* and *Mepe*. All other MCP families had expression profiles that varied in intensity among their respective members, although more MCP genes were upregulated than downregulated. The most downregulated gene was the TN member, *Tnn*. Analysis of the Fibrotic phase of UUO-induced injury (day 8; Fig. 3C,D) had nearly all MCP genes with upregulated expression, while 3 genes exhibited undetectable expression (*Spock1*, *Tnr*, *Mepe*) and 6 genes had downregulated expression (*Smoc1, Spock3*, *Comp, Tnn, Tnxb, Wisp3*). In fact, 5 genes had an expression greater than a 10-fold difference (*Thbs2, Thbs4*, *Wisp1, Wisp2, Spp1*). The SIBLING family was the only one with members having undetectable mRNA levels from normal tissue (Fig. 3B,D). Of these genes, only *Dspp* and *Dmp1* showed distinct upregulation over time while *Mepe* was undetectable in both Acute and Fibrotic UUO-injury.

Differentially expressed MCP genes during the progression of renal fibrosis were identified by DESeq analysis with an adjusted p-value ≤ 0.05 and log2 fold-change > 1.5, or those expressed only after injury with an absolute cut-off FPKM value of >2. From the total 29 MCP genes, 14 were differentially upregulated, with 11 in the Fibrotic stage and 3 in both Acute and Fibrotic stages (Fig. 3E). No genes showed a significant difference in expression that was exclusive to only the Acute phase of a UUO response. In contrast, *Tnn*, *Spock3* and *Comp* were the only MCP genes found to be significantly downregulated (Fig. 3F). *Tnn* was significantly downregulated in both the Acute and Fibrotic phases of injury, whereas *Spock3* and *Comp* downregulations were restricted to the Fibrotic stage. One striking aspect of the UUO model was the number of differentially upregulated genes found only in the Fibrotic stage of injury as compared to the Acute stage, indicating distinct temporal expression. The SPARC family had the highest number of significantly upregulated genes (4 members). The expression for essentially all MCP genes manifested an increasing trend from an Acute to a Fibrotic injury response, where *Tnn* was the lowest expressed gene and *Spp1* was the highest expressed gene of all MCPs from both time points.

### Comparing MCP expression profiles between kidney injury mouse models at different stages of injury

To determine if MCP expression is restricted to a specific mechanistic model of injury or can be generalized irrespective of the initial insult, the mRNA expression levels of MCPs from the FA and UUO models were compared. Differentially expressed genes, either statistically by a DESeq adjusted p-value ≤ 0.05 or with an absolute value > 2, at similar timepoints were compared between injury models: 2-days FA vs. 2-days UUO for an Acute injury analysis, and 7-days FA vs. 8-days UUO for a Fibrotic injury analysis.

**Figure 4:**
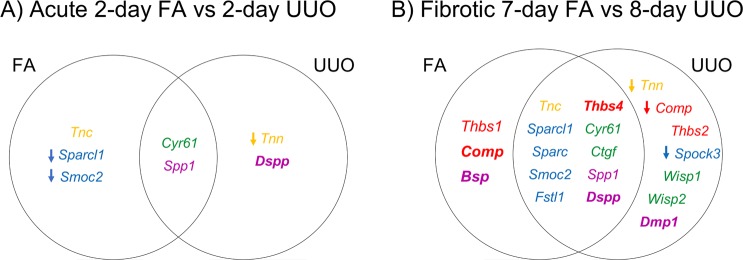
Differential expression of MCP mRNA at different time-points between FA- and UUO-induced injuries. Candidate MCP genes with significant expression found in either the (**A)** Acute phase or (**B)** Fibrotic phase were compared between FA and UUO injuries. Listed genes were considered to be differentially expressed if they had a DESeq analysis with an adjusted p-value ≤ 0.05 and log2 fold-change > 1.5, or an absolute FPKM value > 2 when controls had no detection (**bold** and *italicized*). All genes are upregulated unless specified with a downward arrow to indicate downregulation.

In the Acute phase, we identified differential expression of 3 MCP genes specific to the FA model (*Tnc, Sparcl1, Smoc2*), 2 specific to the UUO model (*Tnn, Dspp*) and 2 overlapping in both models (*Cyr61, Spp1*) (Fig. 4A). However, the Fibrotic phase had many more MCP genes differentially expressed within both injury stimuli (Fig. 4B). A limited number of differentially expressed genes was found in only one of the two injury models. We identified only 2 MCP genes (*Thbs1, Bsp*) specific to FA injury, whereas 6 MCP genes (*Tnn, Thbs2, Spock3, Wisp1, Wisp2, Dmp1*) were specific to the UUO model. Although *Comp* expression was differentially expressed in each injury, the type of expression was injury-specific since it was upregulated in FA while downregulated in UUO. Overall, most differentially expressed genes in the Fibrotic phase were expressed in both models (10 total; *Tnc, Thbs4, Sparcl1, Sparc, Smoc2, Fstl1, Cyr61, Ctgf, Spp1, Dspp*), indicating a conserved MCP gene signature irrespective of the type of injury mechanism. All MCP genes that are differentially expressed in both Fibrotic phases of FA and UUO have the same rising trend when starting from day 2 of the Acute phase, except for *Spp1* which decreases in the FA model from the Acute to Fibrotic phase.

### Integrated analysis of differentially expressed MCP genes in kidney fibrosis

**Figure 5:**
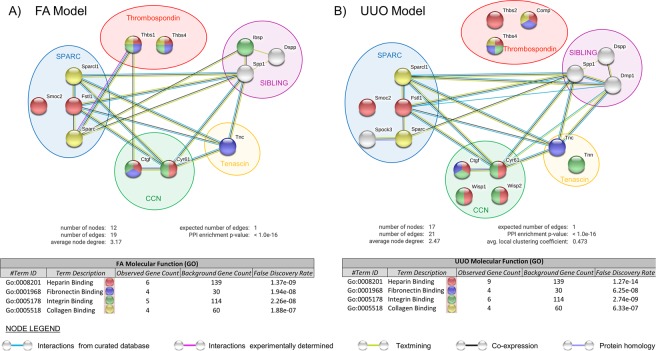
Interaction analysis of differentially expressed MCP genes within the fibrotic phase of kidney injury using the database STRING. Identification of interaction networks and top predicted molecular functions between our candidate MCP genes that were differentially expressed in the fibrotic phase of the (**A)** FA (7-day) and (**B)** UUO injury (8-day) models, using the search tool STRING. Using the Cluster profiler R package, listed genes were considered differentially expressed since they had either a DESeq analysis with an adjusted p-value ≤ 0.05 and log2 fold-change > 1.5, or an absolute FPKM value > 2 when controls had no detection. Colour codes of nodes are based on the annotation term for each “Molecular Function”, and interaction edges are based on a minimal confidence of 0.7 using sources of textmining, experiments, databases and co-expressions.

Bioinformatics analysis of our datasets was performed using the STRING database^25^, which generates a network to summarize predicted associations between MCPs of each injury group with a minimal confidence level of 0.70 (Fig. 5). From the total of 29 known MCP genes, the Fibrotic phases of FA (7-day) and UUO (8-day) experienced a larger number of differentially expressed genes than their respective Acute phases (Fig. 4). STRING was carried out only on this larger set of differentially expressed genes in order to increase the number of predicted associations. From our generated network, MCPs are represented as nodes (Fig. 5; spheres), while their predicted functional associations are represented as edges (Fig. 5; lines). Given the large number of shared MCP genes between UUO and FA, their analysis had created a similar network of edges (Fig. 5). Interestingly, the injury-specific genes for UUO and FA did not substantially create new edges with their shared network, which consequently gave a similar total number of edges between UUO and FA. Taken together, this shared interactome of MCP genes between injuries implies that it may be part of a common fibrotic response that is irrespective of the etiology.

MCP genes were also associated into structured networks of “Molecular Function” as generated by STRING and Gene Ontology (GO). Apart from predicting the interactome between MCPs for each kidney injury, our analysis also predicted the top “Molecular Function” networks from amongst our curated MCPs (Fig. 5; represented by node color). All predicted “Molecular Function” networks involved binding to a particular ECM molecule (i.e. heparin, fibronectin, integrin and collagen), suggesting the possibility of a binding scaffold amongst MCPs when bound to their respective ECM. Although the predicted top annotations did not differ between our kidney injury models, we did reveal differences in the actual MCPs that constituted each “Molecular Function” network.

As expected, our STRING and GO analyses of differentially expressed MCP genes from the FA and UUO models predicted with high relevance the binding to various ECM proteins. In order to gain a generalized functional understanding of MCP genes within the context of their co-expressed ECM microenvironment during the Fibrotic phase, differentially co-expressed MCP (Fig. 4B) and ECM genes from the UUO or FA RNA-Seq dataset were compiled into STRING and GO analysis. For each kidney injury, the combined MCP and ECM dataset permitted us to identify other potentially important interaction networks, and predict with more certainty the top “Biological Processes” from amongst the many that compose Fibrosis (Supplementary Fig. 1). Not surprisingly, the top annotation cluster (represented by node color) for both injuries was “Extracellular Structure Organization”, though the UUO model had 5 MCP genes (*Dmp1, Comp, Smoc2, Ctgf* and *Cyr61*)/ 55 ECM genes while FA had 4 MCP genes (*Ctgf, Smoc2, Bsp2* and *Cyr61*)/ 24 ECM genes. Both injury models also shared top clusters for “Angiogenesis”, “Regulation of Cell Migration” and “Regulation of Cell Adhesion” with the exception that UUO had a larger set of ECM genes. The major difference between both injuries was the additional network that FA had over UUO, which was “Regulation of Peptidase Activity”. This additional network may be necessary during the remission period in the late Fibrotic FA phase when ECM deposits undergo remodeling by peptidase activity, a transition that is not achieved by UUO because of its continual injury-induced ECM production. Understandably, such top “Biological Processes” are typically involved in repair; however, our analysis also provides a cluster of MCP and ECM proteins constituting each “Biological Process” network. These clusters are possibly working together within their respective intricate unit to establish their predicted “Biological Process” network. This information was combined with their interaction network (represented by interaction edges) to expose a rich source of information potentially implicated within the Fibrotic phase of their respective injury models. For instance, combining the results of our predicted “Biological Processes” with that of our various interactomes consequently reveals other probable biological processes that some molecules may have through their association with interacting members, especially for those unclassified MCP molecules (ie. Supplementary Fig. 1; white nodes). Therefore, for each kidney injury we provide specific networks of MCP and ECM molecules sharing various annotation clusters, and reveal the interaction networks between those statistically significant MCP and ECM proteins within the Fibrotic phase.

Overall, the differential expression of such genes may be influencing our identified pathways, leading to the predicted functional changes and disease development which are commonly observed in fibrosis.

### Validating MCP expression within acute and fibrotic phases and correlating them with renal pathology

The RNA-Seq data from both UUO and FA injury models were validated at the level of protein expression by selecting one of the highest changes in mRNA expression profiles between both models from their Acute and Fibrotic phases. As a result, we selected CYR61 and SPP1 to represent Acute MCPs while SMOC2 and FSTL1 were selected to represent Fibrotic MCPs. Such MCP candidates were also selected for their known roles in kidney fibrosis; hence, serving as positive controls of fibrotic MCPs (Table 1). Alternatively, THBS2 was chosen for its novelty in kidney fibrosis, while BSP and DSPP were selected to validate novel MCPs in fibrosis. We performed 250 mg/kg FA or vehicle injections in C57BL/6 J mice then harvested kidneys after 3-, 5- and 7-days. We also performed UUO on C57BL/6J mice and harvested their kidneys after 2- and 8-days post-UUO surgery, using the contralateral kidneys as controls.

**Figure 6:**
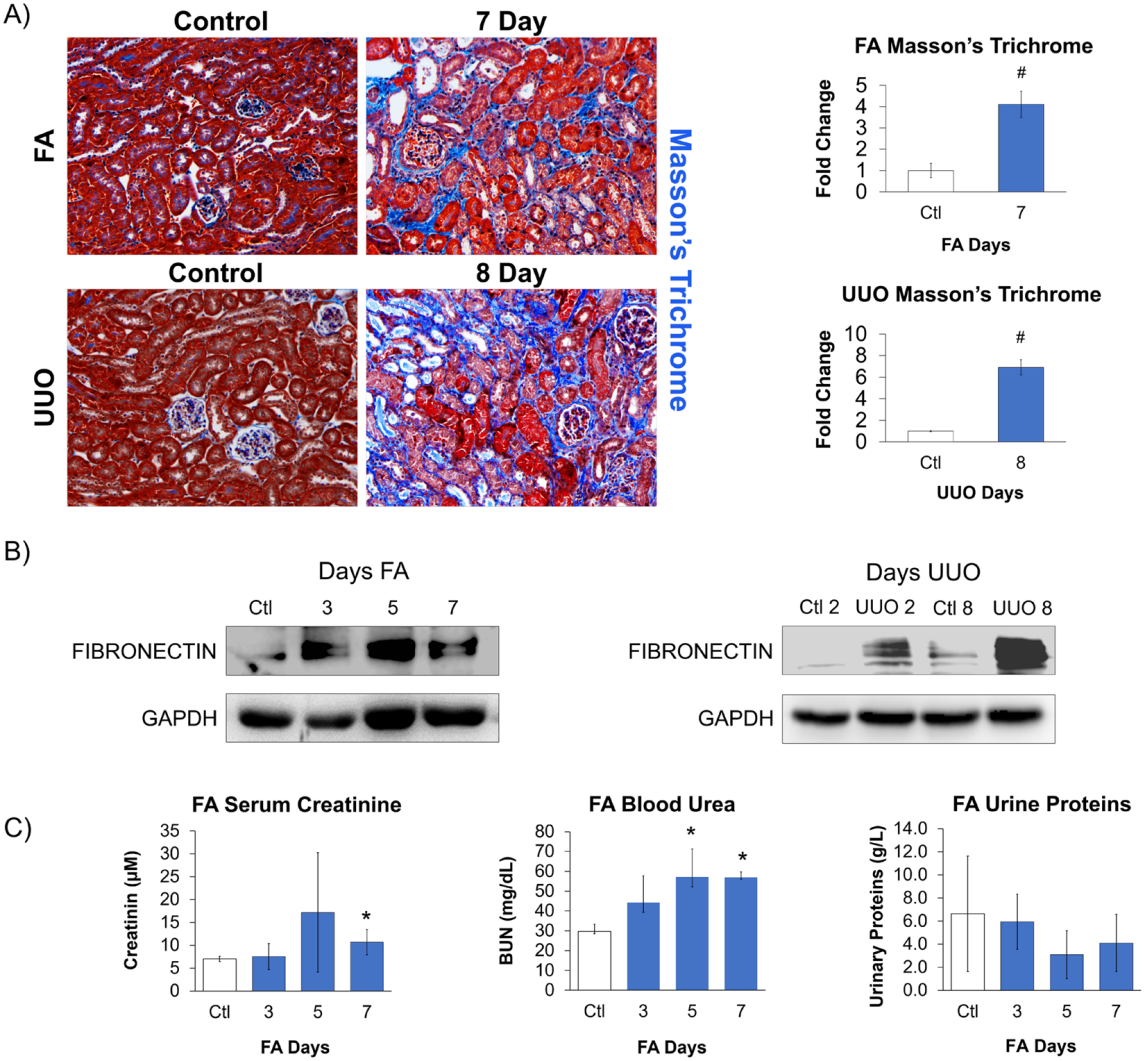
Confirmation of kidney injury within mouse models. Mice were subjected to FA or UUO kidney injury, and kidneys were harvested for **(A)** Masson’s trichrome staining and (**B)** Western Blot analysis for fibronectin to confirm kidney injury. Images are representative of Masson’s trichrome staining (3–5 visual fields/tissue sample) and Western Blots. Masson’s trichrome was expressed as a fold change relative to their respective controls. (**C)** Kidney function was evaluated in the FA mouse model by measuring serum creatinine, blood urea nitrogen (BUN) and urine protein at time points 3-, 5- and 7-days post FA administration. FA and UUO mice were the same used as in Fig. 7. Compared to their respective controls, statistical significance was determined using a *t* test with a Bonferroni correction; #p ≤ 0.017, n = 3; and *p ≤ 0.0125, n = 4.

FA- and UUO-induced fibrosis in the kidney was confirmed by a significant increase in pathological tubulointerstitial fibrosis, as detected by Masson’s trichrome (Fig. 6A), and the classical injury marker, fibronectin (Fig. 6B). As expected, progressive injury in UUO exhibited increasing fibronectin over time, while the regressive FA model reached peak fibronectin expression followed by a recovery period. Since the ureter was tied in the UUO model, clearance from the damaged kidney to evaluate kidney function could only be performed in the FA model. Furthermore, plasma creatinine cannot measure kidney function of the UUO kidney because the unobstructed contralateral kidney compensates any loss in kidney function from the obstructed kidney^26^. Due to these limitations in the UUO model, renal function was only analyzed in the FA model, from which urine and serum were collected to confirm kidney injury by measuring serum creatinine, blood urea nitrogen (BUN) and urine protein (Fig. 6C). Our results show that the FA model had an effect on serum creatinine and BUN, implicating a deterioration in kidney function. However, we did not see a clear effect in proteinuria possibly because FA targets the distal tubule^27^, which is not involved in protein reabsorption; hence, we would not expect altered protein levels. Therefore, kidney functional analysis on FA-treated kidneys clearly showed reduced kidney activity with subsequent recovery, a reflection of it being a regression model.

**Figure 7:**
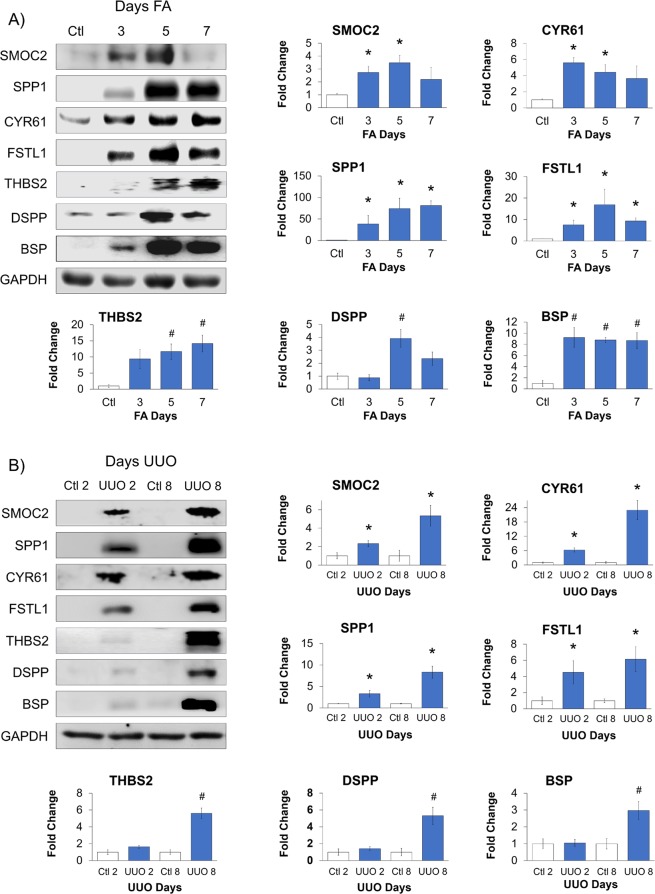
Validating RNA-Seq data of selected MCPs from kidney injury models. Mice were subjected to (**A)** FA or (**B)** UUO kidney injury and lysates were tested for selected candidate MCPs SMOC2, SPP1, CYR61, FSTL1, THBS2, DSPP and BSP. Western Blots are representative images with accompanying quantification analysis of n = 3–4 mice. Each blot was normalized to GAPDH and expressed as a fold change compared to (**A)** FA control (Ctl), (**B)** day 2 control (Ctl 2) vs day 2 UUO (UUO 2) or day 8 control (Ctl 8) vs day 8 UUO (UUO 8). Statistical significance was determined using a *t* test with a Bonferroni correction; #p ≤ 0.017, n = 3; and *p ≤ 0.0125, n = 4.

Western Blot analysis of both injury models revealed that the expression of all of our candidate MCP genes were significantly elevated over the course of injury, irrespective of whether it was initiated by FA administration (Fig. 7A) or UUO (Fig. 7B). Interestingly, all candidate MCPs mirrored the severity of the injury, where MCP expression followed the typical injury regressive and progressive patterns of FA (Figs 6A–C and 7A) and UUO (Figs 6A,B and 7B), respectively.

## Discussion

Current diagnostics and treatments for CKD are not effective for monitoring and preventing progression of the disease. Irrespective of etiology, the majority of CKDs share the pathological feature of excessive ECM deposition, which replaces the flexible parenchymal tissue in kidney, leading to progressive loss of function. Although histology-based testing for ECM deposition represents the gold standard for CKD diagnosis, this painful and invasive procedure, carrying increased risk of complications such as hemorrhaging, is rarely used. We postulated that factors in the extracellular space which process ECM proteins are strongly implicated in a fibrotic pathology. If so, such factors may represent strategic targets towards impeding and/or reversing fibrosis but also allow an improved level of accessibility for therapeutic entry and detection of fibrotic markers. Since MCPs meet these criteria and exhibit specific expression during tissue remodeling processes (i.e. fibrosis), the current study was designed to evaluate by RNA-Seq the dynamic expression of all known members comprising 5 well-characterized MCP families (SPARC, CCN, THBS, SIBLING and TN) over the course of Acute to Fibrotic injury in different fibrotic models. The presence of certain MCPs in diseased tissue could potentially serve as novel biomarkers; moreover, their extracellular location may cause them to shed into circulation and potentially serve as non-invasive diagnostic and prognostic biomarkers. Indeed, several MCPs were shown to be present in the urine of patients afflicted with various tissue remodeling diseases. In particular, among our selected MCP candidates, we previously showed that SMOC2 can be recovered in the urine of CKD patients^24^, while SPP1 and CYR61 have been found in urine of renal ischemic reperfusion injured mice^28^ and prostate cancer patients^29^, respectively.

We present herein the gene expression profiles of all known 29 members comprising the well characterized MCP families SPARC, CCN, THBS, SIBLING and TN. Most of these members, to the best of our knowledge, have not been previously investigated in comparison to other MCPs nor with respect to disease progression following UUO and FA injury, and in other cases have never been studied in kidney fibrosis nor fibrosis in general (Table 1). Of note, 2 members of the SPARC family (*Sparcl1, Smoc1*), 3 from the THBS family (*Thbs2, Thbs3, Thbs4*) and 1 from the CNN family (*Wisp2*) have been studied in the context of fibrosis, but not kidney fibrosis. Furthermore, we report the expression trends of 3 SPARC members (*Spock1, Spock2, Spock3*), 4 TN members (*TnxA, TnxB, Tnr, Tnn*) and 4 SIBLING members (*Bsp, Dmp1, Dspp, Mepe*) which have not been previously described for any type of fibrotic injury.

Among MCPs never studied in fibrosis, *Spock3, Dmp1* and *Tnn* from the UUO model, *Bsp* from the FA model and *Dspp* from both models were the only ones showing differential expression after injury. *Spock3* is known to inhibit the activity of matrix metalloproteinases^30^, a large family of proteases strongly implicated in degrading a variety of ECM proteins that regulate tissue remodeling during repair. *Spock3* has also been shown to be expressed by myofibroblasts, i.e. the effector cells of late stage fibrosis^31^. *Dmp1* has been studied in regulating nucleation of hydroxyapatite, which may have an effect on myofibroblast activity since hydroxyapatite drives myofibroblast activation^32,33^. Less is known regarding *Tnn* whose functions vary with cell type. Indeed this factor inhibits proliferation and differentiation of proteoblasts^34^ but stimulates tumour angiogenesis by elongation, migration and sprouting of endothelial cells^35^. Both *Bsp* and *Dspp* are major structural proteins of the bone matrix, but do not exert any known function in fibrosis, and there is only limited information regarding their tissue localization^36,37^. Of all the MCP genes that we analyzed, only *Mepe* exhibited undetectable levels in either injury model, with or without injury. Our newly classified MCPs of kidney fibrosis are promising targets that merit further investigation since each of their functions can possibly provide new mechanisms of Acute and/or Fibrotic kidney injury.

The FA and UUO models differ considerably in their pathological mechanisms, making them ideal models to determine whether MCP expression signatures are conserved between injuries or otherwise unique to each etiology. Compared to the Acute time period, we only analyzed the Fibrotic stage of both injuries since it had a higher amount of differentially expressed MCP genes that could give a more comprehensive analysis. Although we identified differentially expressed genes that were injury-specific or overlapping between injuries, most of the 29 MCP genes showed similar expression trends between the Fibrotic phases of FA and UUO, except for *Bsp, Wisp1* and *Dmp1* (Figs 2C,D and 3C,D)*. Bsp* showed differential expression in FA but negligible expression in UUO, while both *Wisp1* and *Dmp1* showed differential expression in UUO but downregulation and very low expression in FA, respectively. As previously mentioned, *Bsp* and *Dmp1* have never been studied in fibrosis; however, *Wisp1* is known to induce collagen and fibronectin expression in podocytes^38^, and its serum levels showed potential as a non-invasive biomarker of renal fibrosis in patients with CKD^39^. Overall, our comparative analysis describes differentially expressed MCPs specific to different types of injury, i.e. progressive (UUO) versus a regressive (FA), but also suggests that those MCPs differentially expressed in both FA and UUO could potentially serve as a common therapeutic target and/or biomarker of fibrosis development irrespective of the initial injury. In fact, two FA and UUO overlapping MCPs, *Cyr61* and *Smoc2*, have been independently shown for their therapeutic potential in various models of kidney fibrosis^24,40,41^.

Our STRING analysis of MCP and ECM genes differentially expressed during the Fibrotic phase identified the top predicted molecular functions and biological processes from the many that are known within the context of repair. The shared MCP expression profile between both injuries (Fig. 4B) made up most of these predicted fibrotic functions (Fig. 5 and Supplementary Fig. 1), further suggesting that this conserved set of MCP genes may be implicated in a general pathological mechanism of kidney fibrosis regardless of the nature of injury. As such, it would be worthwhile to study these shared MCPs as possible therapeutic targets in combination, given the unlikelihood that targeting a single MCP would lead to significant inhibition of general fibrotic features.

Candidates for validating MCP expression were selected from those exhibiting the highest changes in mRNA expression in each of the Acute or Fibrotic phases of both injury models. Specifically, CYR61 and SPP1, and SMOC2 and FSTL1, were selected to represent Acute and Fibrotic stage MCPs, respectively. Such MCP candidates were also selected to serve as MCP positive markers of kidney fibrosis (Table 1). SMOC2 was previously implicated in fibrosis from both FA and UUO models^24^. In the current study, SMOC2 protein expression from our FA and UUO models gave similar expression as we previously showed using the same injury models^24^. However, in line with our previous results, we now reveal that SMOC2 expression also correlates with kidney malfunction. Furthermore, SMOC2 has been previously shown to influence the transformation of kidney fibroblasts into myofibroblasts^24^. Although FSTL1, SPP1 and CYR61 were studied in various kidney injury models, herein we present their expression for the first time in the FA model. In a UUO-mouse model study^42^, FSTL1 was shown to be specifically upregulated in kidney myofibroblasts after 2- and 5-days post-surgery, while our analysis was performed in whole kidney. We note however that this factor was shown to be involved in the fibrotic process in other organ models^43,44^, and in one case to influence the pro-fibrotic factor TGFβ during bleomycin-induced pulmonary fibrosis^45^. In the kidney injury models of UUO and ischemia reperfusion (I/R), CYR61 has been closely linked with proinflammatory and fibrotic roles in the early phases of injury, and its inhibition had an antifibrotic effect that could not be sustained over time^46,47^. SPP1 has been extensively shown to be implicated in the early inflammatory phase of repair in many mouse kidney injury models, including UUO, nephrotoxic serum nephritis and diabetic nephropathy^48–50^. We also selected candidates for their novelty in fibrosis or kidney fibrosis. Therefore, we selected THBS2 since it has never been studied in the context of kidney fibrosis, while we present for the first time the expression of BSP and DSPP in a fibrotic setting.

For most of our candidate MCPs, protein levels correlated with mRNA expression, and also with the pattern of injury progression in UUO and injury regression in FA, which were evaluated using fibronectin. Furthermore, we observed that the expression of MCP candidates correlated with the deterioration of kidney function and with Masson’s Trichrome staining of fibrosis. Despite these correlating results of our MCP candidates, there was a discrepancy between SMOC2 mRNA and protein expression in our FA but not UUO model. Indeed, we expected high levels of SMOC2 mRNA within the Fibrotic phase to translate into similar protein levels; however, we observed the highest protein expression in the Acute phase. This could imply a regulatory mechanism affecting protein expression, such as mechanisms of translational and/or posttranslational regulation.

In conclusion, we have provided an outline of MCP gene expression patterns at various stages of kidney injury development, with predicted functional networks that correlate with, and potentially mediate, fibrotic progression. Our analysis reveals potential candidates for targeted therapeutics, as well as promising biomarkers with early diagnostic and prognostic value.

## Methods

### Chemicals

Folic Acid (FA), Ponceau S, Ammonium Persulfate and TEMED were products of Sigma-Aldrich CAN. Tween20, Tris-base, Glycine, EDTA, Methanol, SDS, Acrylamide and Bis-acrylamide were obtained from VWR. All chemicals were of ACS grade or higher. Trimidox and Torbugesic were obtained from CDMV, Saint-Hyacinthe, CAN.

### Animal models

C57Bl/6J mice (The Jackson Laboratory, USA), aged from 8 to 12 weeks, were housed in the animal facility at the Hôpital Maisonneuve-Rosemont (HMR) Research Center and were provided with Harlan Teklad rodent diet #2018 (Envigo, Lachine, CAN) and water ad libitum. All experiments were conducted according to the Canadian Council on Animal Care guidelines for the care and use of laboratory animals, and under the supervision and approval of our local animal care committee, Comité de protection des animaux du CIUSSS de l’Est-de-l’ile-de-Montréal (Approved Protocol #2018-1261).

### Surgery and experimental protocol

Two mouse models of kidney fibrosis were used as previously described^24^. Surgeries and injections were consistently performed at the same time of day. These models are briefly described:

FA Model. Male C57Bl/6J mice (25–29 g) aged from 8 to 12 weeks received a single i.p. injection of FA (250 mg/kg) dissolved in a 0.3 M sodium bicarbonate solution. The day before sacrifice, urine was collected for 24 h in metabolic cages to determine urine protein concentration. Mice were euthanized 3-, 5-, and 7-days following FA administration, for organ and blood collection. Euthanasia was performed under isoflurane anesthesia.

UUO model. Male C57Bl/6J aged from 8 to 12 weeks were anesthetized by isoflurane inhalation, and their left kidney was exposed by flank incision. The left ureter was ligated at two points proximal to the kidney with 3–0 sutures (Ethicon; Perma-hand Silk/Black Braided). The left kidney was used as a contralateral control. Mice received fluid lost replacement (1 ml normal saline, heated at 37 °C, i.p.) and antibiotics immediately after surgery (Trimidox). Mice were euthanized at 2- and 8-days following surgery for organ and blood collection. Euthanasia was performed under isoflurane anesthesia.

In both models, at the time of sacrifice, kidneys were perfused with cold PBS, immediately excised and rinsed in phosphate buffered saline (PBS). Samples for histological staining were fixed in formalin for 24 to 48 hours and embedded in paraffin. Samples for Western Blot were flash-frozen in liquid nitrogen. Blood was collected for further biochemical characterization.

### Histology

Paraffin-embedded kidneys were cut into 4- to 6-μm sections and processed for Masson’s Trichrome staining. Images were acquired in the Imaging Facility at HMR. All images were analyzed through NIH ImageJ using a color threshold algorithm (identical threshold settings for compared image sets) written by Gabriel Landini (version v1.8) available at http://www.mecourse.com/landinig/software/software.html.

### Biochemical parameters

Creatinine concentration in serum samples was measured with a modified enzymatic assay (CREP2, Roche Diagnostics, Canada). Briefly, samples were prepared by transferring 50 µl of standard or serum to a 1.5 ml microcentrifuge tube. Proteins were precipitated and supernatants were lyophilised on a speed vac (LABCONCO freeze Dry system, VWR, Canada). Lyophilised samples were reconstituted in 25 µl deionised water and vortex mixed thoroughly. After a 30 min incubation at room temperature, samples were vortex mixed thoroughly again and then centrifuged at 11 000 × g for 5 min. 8 µl of each supernatant were transferred to a half area plate (Costar #3695), in duplicate. 62 µl of CREP2 R1 was added to each well. The plate was vortex mixed (MixMate, Eppendorf, Canada) at 1000 rpm 30 sec, and incubated 15 min at 37 °C to allow endogenous creatinine degradation. Readings at 405 nm and 540/630 nm were performed and 31 µl of CREP2 R2 was then added to each well. The plate was vortex mixed at 900 rpm 30 sec. Readings were performed on a kinetic mode, each minute for a 30 minutes period (ELx808, BioTek, USA).

Serum urea was measured with the Quantichrom Urea Assay Kit (BioAssay Systems, Hayward, CA, USA) according to manufacturer’s instructions. Urinary Creatinine and urine proteins were measured on an Architect c16000 clinical chemistry analyzer (Abbott Diagnostics, IL, USA), using a kinetic alkaline picrate method and a turbidimetric method respectively.

### Tissue preparation for western blot analysis

Frozen biopsies of mice kidney were homogenized in RIPA buffer (Pierce, ThermoFisher, Canada) containing 1x protease and phosphatase inhibitor cocktail (Roche, Sigma, Canada) using an overhead stirrer (IKA). Protein concentration was determined using the BCA method (Pierce, ThermoFisher, Canada).

### Western blotting

Protein levels were assessed by Western Blot analysis. Samples of 10 to 50 µg of protein were separated by electrophoresis on a 10% polyacrylamide gel containing 0.4% sodium dodecyl sulfate (SDS) and were electrophoretically transferred onto nitrocellulose membrane (Amersham Protran 0,45 µm, GE Healthcare Life science, Mississauga, Canada). Membranes were saturated with 5% NFDM in Tris buffered saline (TBS) containing 0.1% Tween20 (TBST) and washed with TBST. The following primary antibodies were used to detect the specific protein: anti-SMOC2 (1:200; R&D Systems, AF5140), anti-CYR61 (1:2,000; aka CCN1 R&D Systems, AF4055), anti–FSTL1 (1:10,000; R&D Systems, AF1738), anti-SPP1 (1:10,000; aka OPN R&D Systems, AF808), anti-THBS2 (1:1000; Santa Cruz Biotechnology, sc-136238), anti-DSPP (1:750; Santa Cruz Biotechnology, sc-73632), anti-BSP (1:750; Santa Cruz Biotechnology, sc-73630) and anti-GAPDH (1:5,000; Abcam, ab9485). Horseradish peroxidase–conjugated secondary antibodies against sheep (Sigma, A3415), goat (Sigma, A5420) and rabbit (Santa Cruz Biotechnology, sc-2357) were used to detect the appropriate primary antibody. Bands were detected with the Clarity Max Western ECL Substrate from Bio-Rad Laboratories (Hercules, USA). Results were analyzed by computer-assisted densitometry using ImageQuant LAS-4000 system from GE Healthcare Life Sciences (Mississauga, CAN), ImageJ and FUJIFILM MultiGauge V3.0.

### RNA-Seq database and bioinformatics analysis

We processed RNA-Seq data in the following procedure. Raw read counts from the FA (GSE65267)^11^ and UUO (GSE79443)^12^ mouse models were retrieved from the Gene Expression Omnibus (GEO) database repository (http://www.ncbi.nlm.nih.gov/geo/). Transcripts with a read count lower than 1 in all samples were removed. Differentially expressed (DE) transcripts were identified in each time point using DESeq. 2 package^51^ implemented in R with default arguments. Differentially expressed MCP transcripts were those containing a minimal log-fold change of >1.5 and adjusted p-value ≤ 0.05, or an absolute FPKM value of >2 when controls had no mRNA detection. To identify extracellular molecules, we used the clusterProfiler^52^ R package on only differentially expressed genes (log-fold change of >1.5 and adjusted p-value ≤ 0.05) belonging to the GO term: extracellular space (GO:0005615). Ontology terms with an FDR ≤0.05 were considered significant.

Members of the MCP families were characterized for their known involvement in fibrosis. Searches were performed using the databases PubMed, and Kidney and Urinary Pathway Knowledge Base (KUPKB) for each of our 29 MCP gene candidates in combination with the inclusion key words “fibrosis” or “kidney fibrosis”. Results for each MCP were tabulated as being either involved in fibrosis that includes kidney fibrosis, fibrosis that excludes kidney fibrosis or not involved in any fibrosis.

The Search Tool for the Retrieval of Interacting Genes/Proteins (STRING)^53^ was used to visualize network interactions within our MCP candidate genes based on databases of known and predicted protein interactions. GO was used as a complementary tool within STRING to identify networks of molecular functions and biological processes.

### Statistical analysis

Results are expressed as mean ± standard error. Statistical significance for multiple comparisons was calculated by two-tailed Student’s *t* test with a Bonferroni correction (p ≤ 0.05/n, where n = sample size). Statistical analyses were performed with GraphPad Prism v6.05.

## Supplementary information

Supplementary Information

## Data Availability

The datasets analysed during the current study are available in the Genome Expression Omnibus Database (http://www.ncbi.nlm.nih.gov/geo/). Folic Acid (GEO# GSE65267) and UUO (GEO# GSE79443).
