# Supplementary material for: Characterization of Matricellular Protein Expression Signatures in Mechanistically Diverse Mouse Models of Kidney Injury
Source: Sci Rep. 2019 Nov 13;9:16736. doi: 10.1038/s41598-019-52961-5 (PMC6854083; doi:10.1038/s41598-019-52961-5)

Characterization of Matricellular Protein Expression Signatures in Mechanistically Diverse Mouse Models of Kidney Injury

Daniel Feng[1,2#], Cindy Ngov[3#], Nathalie Henley[2], Nadia Boufaied[4] and Casimiro Gerarduzzi[1,2,5*]

[1]Département de Pharmacologie et Physiologie, Faculté de Médecine, Université de Montréal, Montréal, Québec, Canada
[2]Centre de recherche de l'Hôpital Maisonneuve-Rosemont, Faculté de Médecine, Centre affilié à l'Université de Montréal, Montréal, Québec, Canada
[3]Department of Microbiology and Immunology, McGill University Health Centre Research Institute, Montréal, Québec, Canada
[4]Division of Urology and Cancer Research Program, McGill University Health Centre Research Institute, Montréal, Québec, Canada
[5]Département de Médecine, Faculté de Médecine, Université de Montréal, Montréal, Québec, Canada

#These authors contributed equally to this work.
*Corresponding author

Running title: Characterizing Matricellular Protein Expression during Kidney Injury

Key words: Chronic Kidney Disease, Acute Injury, Fibrosis, Animal Models, Folic Acid, Unilateral Ureter Obstruction (UUO), RNA-Seq

***Corresponding author:**

Casimiro Gerarduzzi
Division of Nephrology, Maisonneuve-Rosemont Hospital
CIUSSS de l'Est-de-l'Île-de-Montréal
5345, boul. de l'Assomption
Montreal, QC, Canada
H1T 2M4
Tel: 514-252-3400 ext:2813
casimiro.gerarduzzi@umontreal.ca

A)

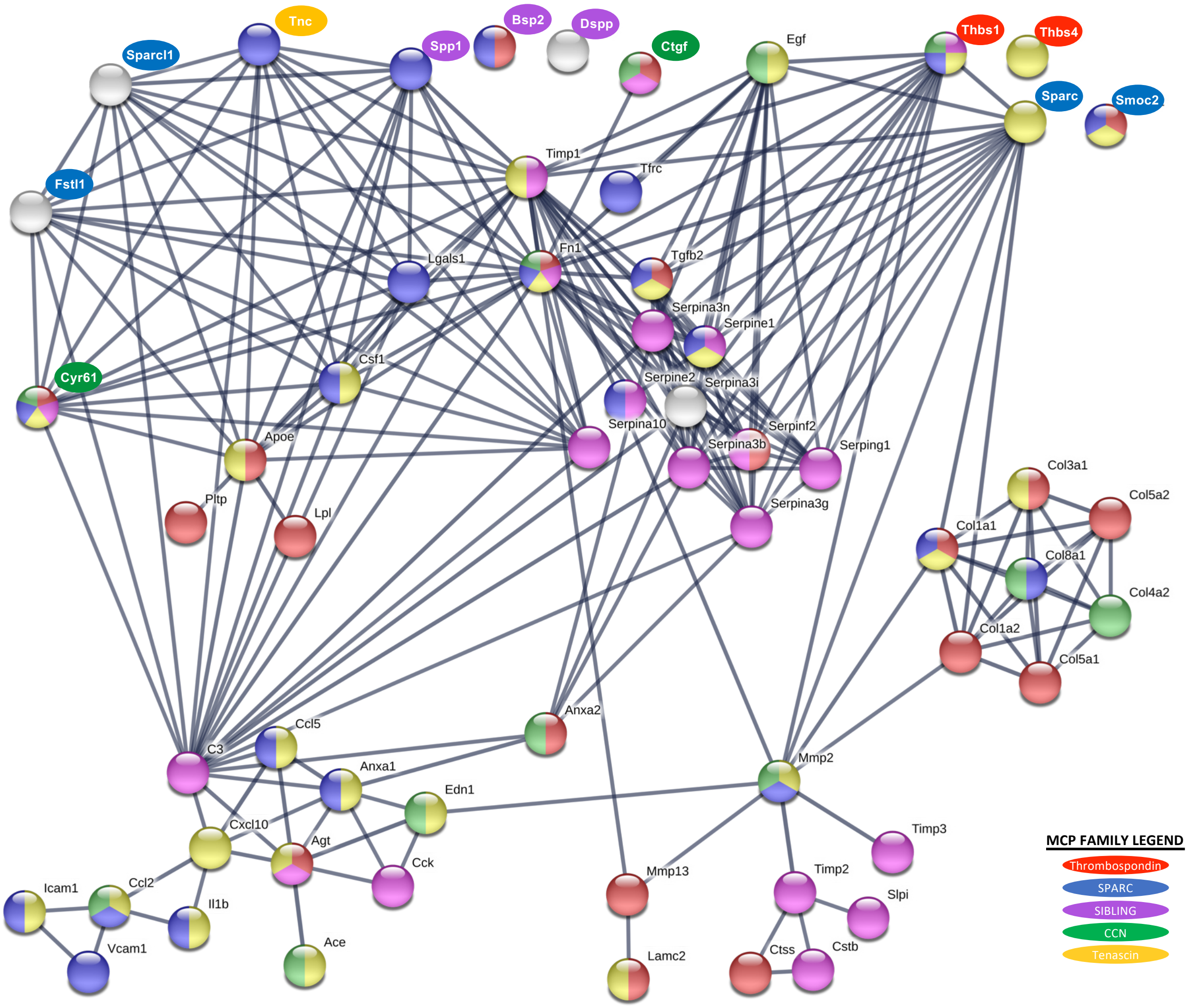

Biological Process (GO)					
#term ID	*term description*	*observed gene count*	*background gene count*	*false discovery rate*	*node color*
GO:0030198	extracellular structure organization	27	214	9.73E-30	
GO:0052547	regulation of peptidase activity	27	416	4.18E-23	
GO:0030334	regulation of cell migration	32	805	4.80E-22	
GO:0030155	regulation of cell adhesion	27	624	1.34E-19	
GO:0001525	angiogenesis	17	303	8.25E-14	
	unclassified in top cluster				

Supplementary Figure 1. **(Continued on next page)**

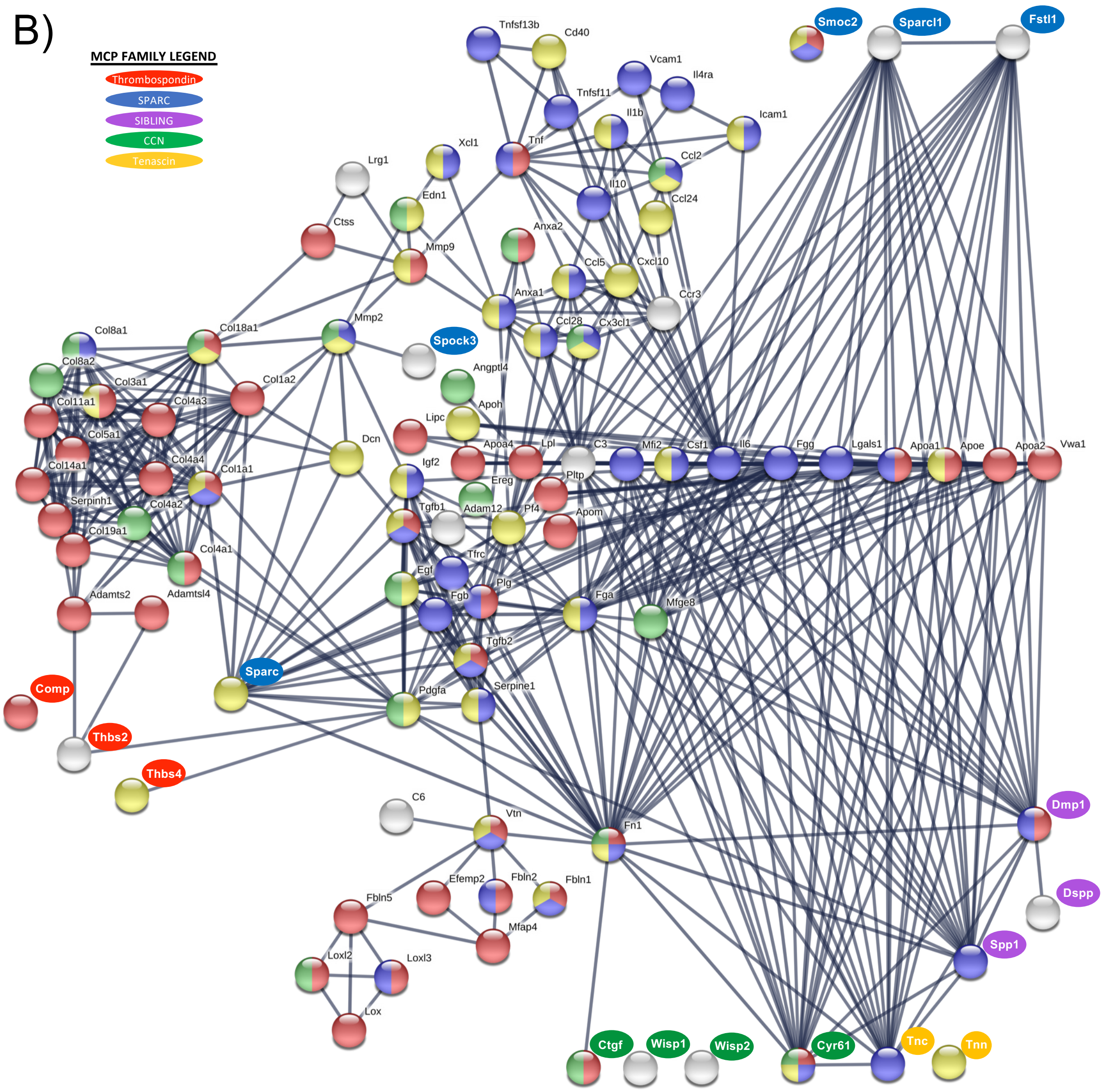

Biological Process (GO)					
#term ID	term description	observed gene count	background gene count	false discovery rate	node color
GO:0030198	extracellular structure organization	60	214	1.43E-75	
GO:0030155	regulation of cell adhesion	53	624	1.76E-41	
GO:0030334	regulation of cell migration	52	805	2.32E-35	
GO:0001525	angiogenesis	30	303	1.18E-24	
	unclassified in top cluster				

Supplementary Figure 1. **(Continued on next page)**

Supplementary Figure 1. Biological processes of differentially expressed MCP and ECM genes within the Fibrotic phase of kidney injury using STRING. The search database STRING was used to identify top functional networks between MCP and ECM genes with expression values that were differentially expressed within the Fibrotic phase of the **A)** FA (7-day) and **B)** UUO injury (8-day) models. Using the Cluster profiler R package, listed genes were considered differentially expressed if they had a DESeq analysis with an adjusted p-value $\leq$0.05 and log2 fold-change $\geq$1.5, or an absolute FPKM value $\geq$2 when controls had no detection. Colour codes of nodes are based on the annotation term for each "Biological Process", and interaction edges are based on a confidence of 0.9 using sources of textmining, experiments, databases and co-expressions. Candidate MCP genes are within a coloured ellipse while ECM genes are in black font.

Original Western Blots

Figure 6B Folic Acid

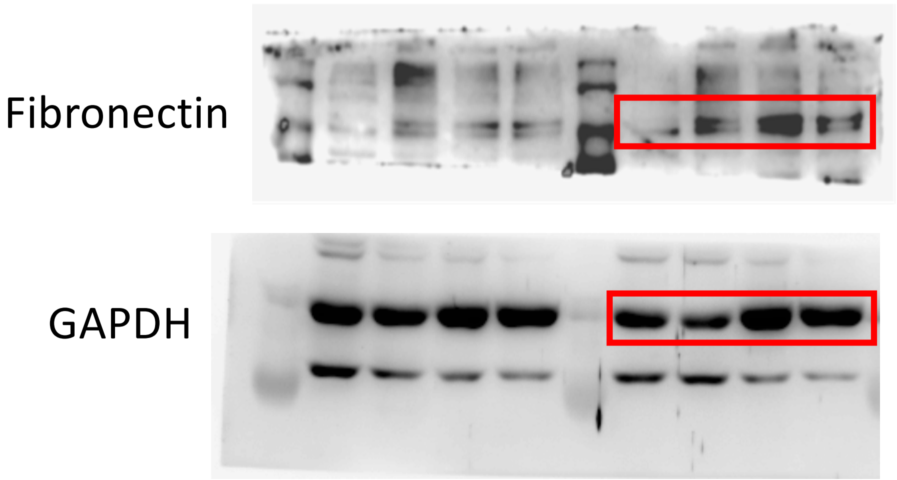

Figure 6B Unilateral Ureteral Obstruction

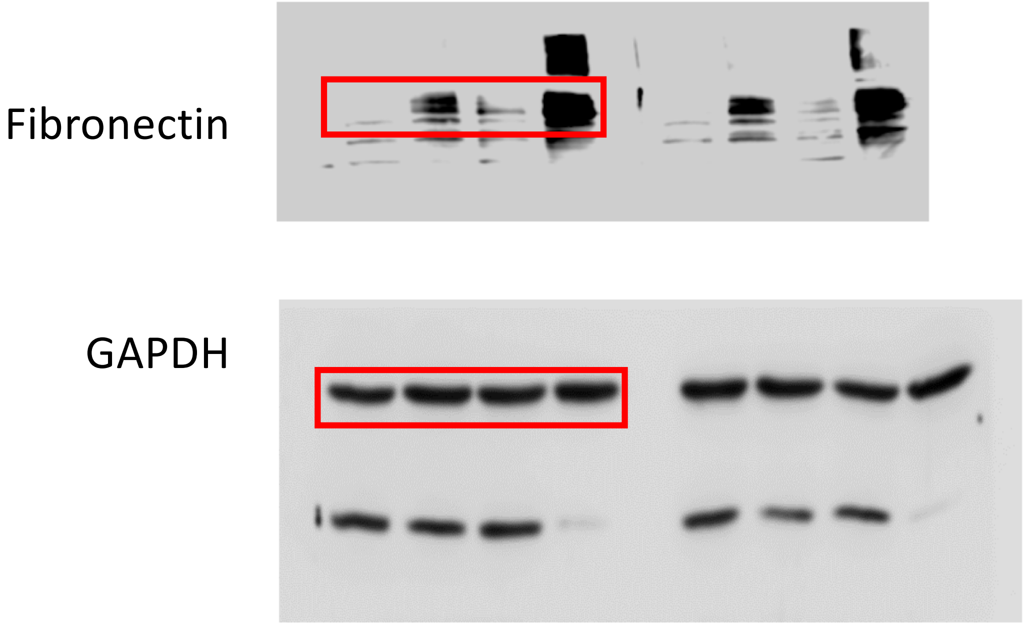

Figure 7A Folic Acid

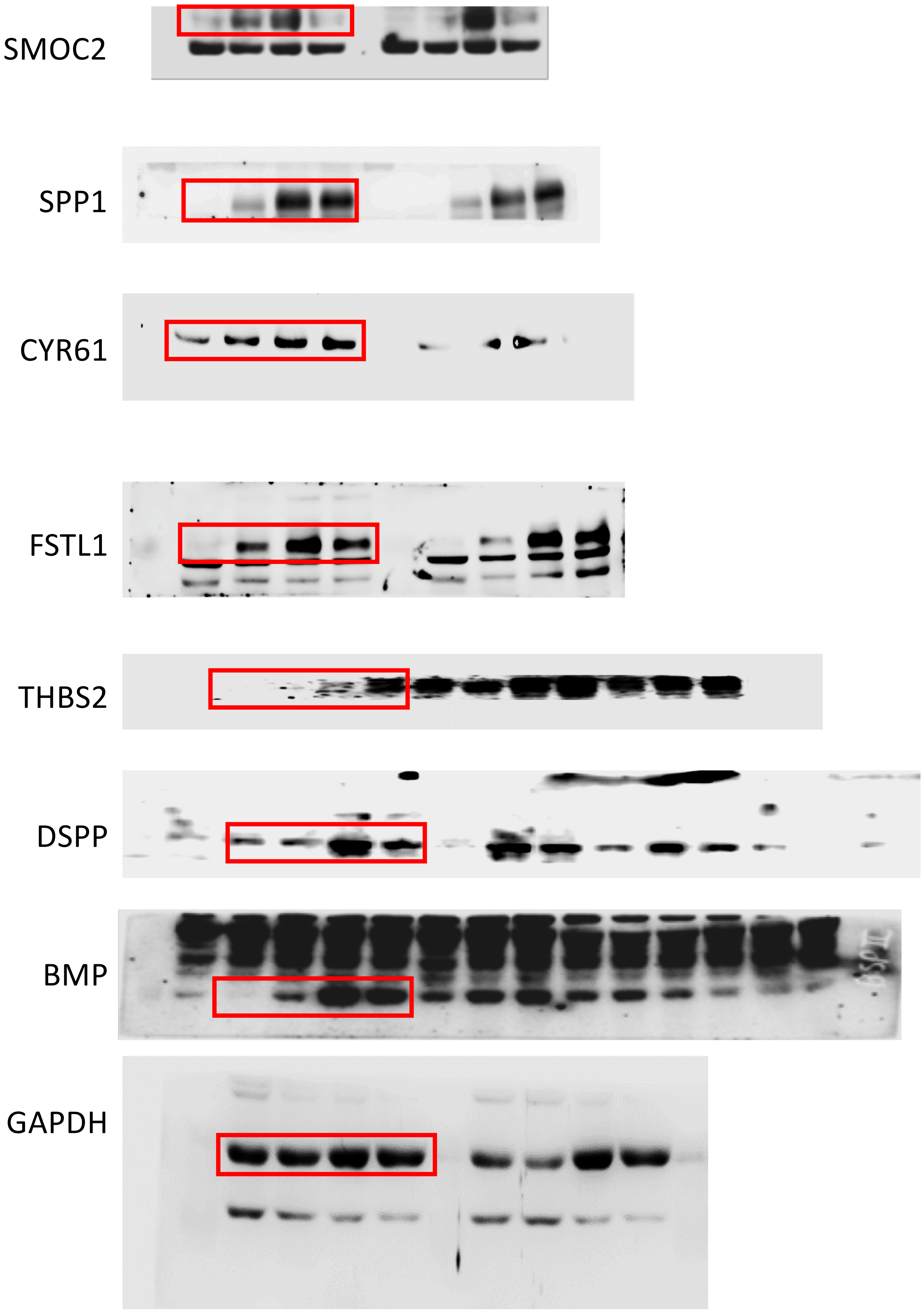

Figure 7B Unilateral Ureteral Obstruction

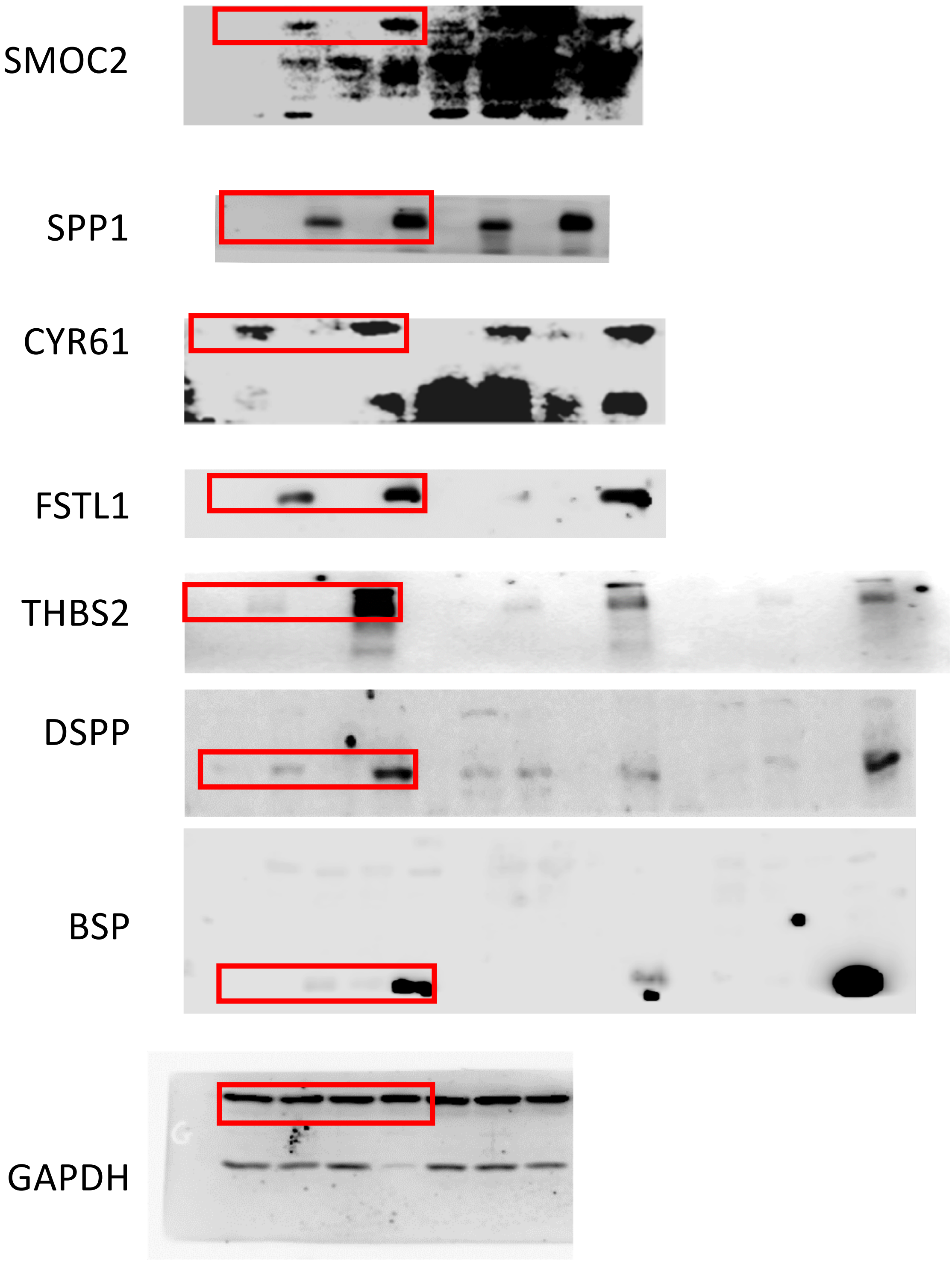

Supplement: Supplementary file 1 — Supplementary Information [file 41598_2019_52961_MOESM1_ESM.pdf]
